# Investigations on the Impact of Material-Integrated Sensors with the Help of FEM-Based Modeling

**DOI:** 10.3390/s150202336

**Published:** 2015-01-22

**Authors:** Gerrit Dumstorff, Walter Lang

**Affiliations:** Institute of Microsensors, -Actuators, and -Systems, Microsystems Center Bremen, University of Bremen, 28359 Bremen, Germany; E-Mail: wlang@imsas.uni-bremen.de

**Keywords:** sensor integration, material-integrated sensing, FEM-based model, effect of a wound, *μ*-hybridization, function scale integration

## Abstract

We present investigations on the impact of material-integrated sensors with the help of finite element-based modeling. A sensor (inlay) integrated with a material (matrix) is always a foreign body in the material, which can lead to a “wound effect”, that is degradation of the macroscopic behavior of a material. By analyzing the inlay's impact on the material in terms of mechanical load, heat conduction, stress during integration and other impacts of integration, this wound effect is analyzed. For the mechanical load, we found out that the inlay has to be at least as stretchable and bendable as the matrix. If there is a high thermal load during integration, the coefficients of the thermal expansion of the inlay have to be matched to the matrix. In the case of a high thermal load during operation, the inlay has to be as thin as possible or its thermal conductivity has to be adapted to the thermal conductivity of the matrix. To have a general view of things, the results are dimensionless and independent of the geometry. In each section, the results are illustrated by examples. Based on all of the results, we present our idea for the fabrication of future material-integrated sensors.

## 1. Introduction

Getting measurement quantities out of a material is of significant interest when stating its physical condition. Typical applications in which this is necessary are structural health monitoring, condition monitoring or *in situ* process measurement. Over the past few years, two approaches had been made to get real-time knowledge about what is happening in the material:

- Surface-mounted sensors;
- Material-integrated sensors.

Surface-mounted sensors are mostly based on acoustic measurements. A transmitter emits a wave, which is recorded by a receiver. If the material changes its properties, e.g., cracks appearing in the material due to excessive load, the waveform changes, which can be seen by evaluating the recorded data. Most of the scientific work is not focused on the sensor (transmitter/receiver) itself, but on the algorithms to make the right conclusions on what is happening in the material when the waveform changes [1–3].

In the case of material-integrated sensors, the sensors are brought into the material and merged together with the same. Measurements are done in the material. One of the most common approaches is integrating optical waveguides in the material [4–6]. Expensive optical evaluation, minimal bending radii of the fibers and less measurement categories limit this technique. A second approach is integrating micro-sensors, like strain gauges, thermocouples or capacitive sensors, in a material. Concerning sensor functions, there are several benefits when integrating sensors in a material: The generated data from an integrated sensor describes the physical condition of the material. Better shielding in harsh environments due to full encapsulation is provided. There is also a higher sensitivity of material-integrated strain gauges with respect to surface-mounted devices due to better mechanical coupling [7].

Over the past few years, different sensors have been integrated with different materials by various groups. One of the earliest materials in which a sensor was integrated is concrete. For example, the hydration of concrete was measured [8] or the corrosion in a concrete structure [9] was monitored. In today's research, the most used material for integrating sensors is fiber-reinforced polymers, especially carbon fiber-reinforced plastics (CFRP). This is driven by wing applications for air planes or wind power systems to measure strain [10] or cracks [11–13] in the composite. Monitoring the curing of the epoxy resin of the composite with an interdigital structure is also of high interest [14,15]. There is already a commercial sensor for this [16]. Along with this, the machining of CFRP has been characterized by integrating temperature sensors in the compound [17]. One of the most challenging materials in which to integrate sensors is metals. One focus is the characterization of the machining processes, like grinding [18] or welding [19], to get knowledge about the impact of the machining process on the metal. Another focus is on sensors in aluminum by integrating pieozceramics [20–23], thermogenerators [24] or electrical systems [25] during casting for applications like structural health monitoring. Integrating piezoceramics in aluminum during deep drawing is presented in [26]. Further materials that have been focused on for sensor integration are boron-nitride-ceramics with integrated thermocouples to measure temperature during the machining of a workpiece [27] or a gasket with an integrated strain gauge to measure the deformation [28]. When the sensors are essentially silicon chips, the ultimate tensile or bending strength can dramatically change. This has been presented by the authors for carbon fiber-reinforced plastics [29] and epoxy resin [30], where tensile and bending tests of test specimens with integrated silicon substrates were made. However, whichever material in which to integrate a sensor is focused on, general investigations on the inlays impact on the matrix cannot be found in the literature.

**Figure 1.**
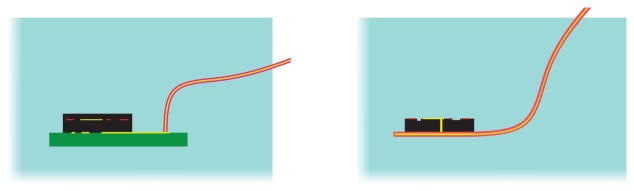
Two different approaches to material-integrated sensors by using common techniques. (**Left**) A sensor chip made of silicon in a housing is mounted on a PCB, and energy and data are provided via a cable; (**Right**) A bare die mounted on a FPCB (flexible printed circuit board), which is also the electrical connection to the outside of the matrix.

Two examples in Figure 1 illustrate two different ways of integrating an inlay in a matrix. Both approaches can be built up with established technologies. In the following, we will name the sensor “inlay” and the material in which the inlay is integrated “matrix”. “i” will indicate the inlay and “m” the matrix. The inlay is based on a silicon chip with functional structures. On the one hand, the chip has a housing and is mounted on a PCB. Energy and data are provided via a cable. On the other hand, the chip is a bare die mounted on a FPCB (flexible printed circuit board). The FPCB leads out of the matrix to provide data and energy. In both approaches many materials with different physical properties are brought into the matrix. This will degrade the macroscopic behavior of the matrix. For example, the ultimate mechanical strength is reduced or, under thermal aspects, the inlay acts as a heat bridge, which might lead to overheating. From a more general view, it can be said that an inlay integrated in the matrix will always have an impact on the matrix. We call this the “effect of the wound”. This leads to the fundamental question: What is the impact of the inlay on the matrix? Our first approach to investigate this question was restricted to tensile and bending load for fixed geometry values [31]. In this paper, we want to proceed towards a detailed analysis of the foreign body effect in terms of mechanical load, thermal management and stress during integration. These are the main loads of the materials and components in real applications in the case of material-integrated sensors for structural health monitoring, condition monitoring or manufacturing monitoring. In some applications, there are other impacts due to integration, like water uptake or radiation, and hence, we will focus on this, too. In contrast to [31], alternating geometries are analyzed to see the influence of the inlay's geometrical parameters. It is our goal to perform general investigations on the impact of the inlay on the matrix, which has not been done yet. From this, we want to derive design rules for the fabrication of material-integrated sensors.

## 2. Mechanical Impact of an Inlay

The most important part when designing inlays is to have a look at the mechanical stress state. It will only be possible to integrate inlays in a matrix when the mechanical influence on the constructive element is reduced to a minimum. To have first a simple view of things, two examples for tensile load and bending load can simply show the challenges:

The law for elastic deformation (Hooke's law) is generally known as:

(1) σ=ϵ×E

where *σ* is the stress generated by a deformation *∊* and the Young's modulus *E*. If we assume that the thickness of the inlay *d_i_* is much smaller than the thickness of the matrix *d_m_* and the inlay and matrix undergo the same tensile load, then:

(2) $$\begin{array}{c} \epsilon_{i}=\epsilon_{m}\\ \frac{E_{i}}{\sigma_{i}}=\frac{E_{m}}{\sigma_{m}}\\ \sigma_{i}=\sigma_{m}\frac{E_{i}}{E_{m}} \end{array}$$

The stress in the inlay depends on the ratio of the Young's moduli of the inlay and matrix. The stress in the inlay in comparison to the stress in the matrix is higher when *E_i_* > *E_m_* and lower when *E_i_* < *E_m_*. A simple example is a piece of silicon (*E*_Si<100>_ = 130 GPa) integrated with polycarbonate (*E*_PC_ = 3 GPa): the stress in the silicon will be around 43-times higher than in the polycarbonate.

While this first approach is only for tensile load, a similar, simple approach can also be made for the bending case. When a bar is bent by a moment *M*, the curvature radius *ϱ* for small elongations is [32]:

(3) $$\varrho =\frac{E\times I}{M}$$

with the area moment of inertia *I*.

If an inlay is integrated in this bent bar, then the inlay has the same curvature as the bar:

(4) $$\varrho_{i}=\varrho_{m}$$

(5) $$\frac{E_{i}\times I_{i}}{M_{i}}=\frac{E_{m}\times I_{m}}{M_{m}}$$

The moment of inertia for a bar with a squared cross-section and an edge length *a* is:

(6) $$I=\frac{a^{3}}{12}$$

If we assume that the inlay has also a squared cross-section and an edge length of 0.1*a*, then Equation (5) is:

(7) $$\frac{E_{i}\times 0.001a^{3}}{12M_{i}}=\frac{E_{m}\times a^{3}}{12M_{m}}$$

(8) $$M_{i}=0.001M_{m}\frac{E_{i}}{E_{m}}$$

For *E_i_* = *E_m_*, the moment bending the inlay is much smaller than the moment bending the bar. Even if the Young's modulus of the inlay is much higher than the matrix (e.g., silicon and polycarbonate, as in the previous section), it has nearly no influence on the bending case. However, Equation (3) implies that the inlay is in the neutral fiber. Integrating a temperature sensor near the neutral fiber can be fruitful, but it will not be useful to integrate a strain sensor near the neutral fiber, because it will not measure any strain. In addition to the mechanical behavior of the inlay and the matrix, the position of the inlay has to be discussed, as well.

**Figure 2.**
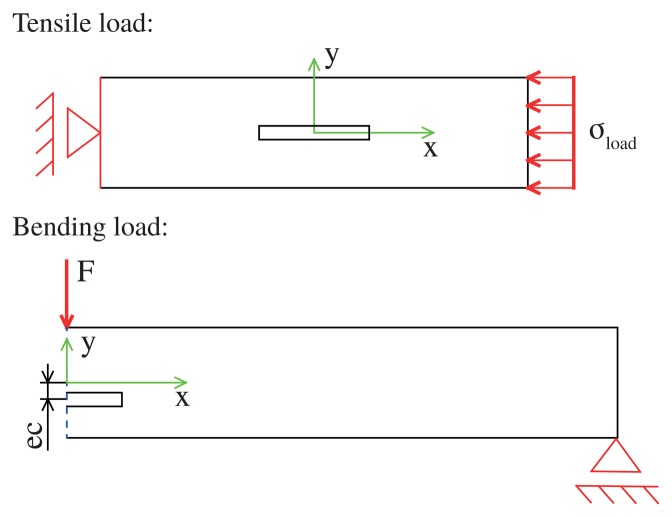
Geometry for the two load cases. For tensile load, the left boundary of the composite was set to a floating bearing, and the right boundary was set to *σ*_ref_. In the case of bending, the right side was set to a floating bearing, while in the middle, a force *F* is bending the bar. The blue dashed line is the symmetry line and *ec* the eccentricity of the inlay.

To have a more detailed view of the mechanical stress state of the inlay and the matrix, a view of the plane stress state is useful. This is done with the help of an FEM simulation for two different load cases: tensile load and bending load. In the model, a composite made of a small bar representing the inlay is integrated with a big bar representing the matrix (see Figure 2). The inlay and the matrix differ in the elastic modulus, but not concerning other material parameters. Intrinsic stress, e.g., caused by the integration process, is neglected. If there are no external loads, both parts are stress free. In the case of linear elastic isotropic material behavior, the ratio of the moduli is:

(9) $$e=\frac{E_{\text{inlay}}}{E_{\text{mamx}}}$$

To have a more suitable view of the stress generated in the inlay and matrix, as well as at the inlay/matrix interface, the use of an equivalent stress is helpful. For different stress states, different equivalent stress hypotheses can be used. A classical hypothesis that is often used is the von Mises stress [33]. For the plane stress state, there are two main stresses *σ_x_* and *σ_y_* and the shear stress *τ_yx_* = *τ_xy_*. The von Mises stress is:

(10) $$\sigma_{\text{von Mises}}=\sqrt{\sigma_{x}^{2}+\sigma_{y}^{2}-\sigma_{x}\sigma_{y}+3\tau_{xy}^{2}}$$

With the help of an FEM simulation, an overview of the reaction forces in the composite is shown. Simulations have been made with Comsol Multiphysics using the solid mechanics module and a stationary study in which:

(11) $$-\nabla \sigma =F_{\text{ext}}$$

is solved. This means that external forces *F*_ext_ change the stress *σ* in the material. The average mesh quality was kept higher than 0.8.

The stress in our simulation is described by the dimensionless stress parameter *q*, which is in dependencyof the stress without an inlay:

(12) $$q=\frac{\sigma_{\text{von Mises}}}{\sigma_{\text{ref}}}$$

For the tensile load *σ*_ref_ = *σ*_load_ and for the bending load, the reference stress *σ*_ref_ is the von Mises stress at the outer edge of the matrix *σ*_outer edge_. *e* (ratio of the moduli) was varied between 0.05 and 20. The Poisson ratio was set to 0.25. The basic geometric parameters of the composite are:

- Tensile load: inlay 0.5 mm × 4mm, matrix 4mm × 20mm;
- Bending load: inlay 0.5mm × 4mm, matrix 4mm × 100 mm.

Since in the bending case there is symmetry to the y-axis, only half of the geometry has been modeled. All geometrical parameters were freely selected. In all simulations, we varied the geometrical parameters. All results are dimensionless and independent of the geometry.

### 2.1. Mechanical Impact at Tensile Load

**Figure 3.**
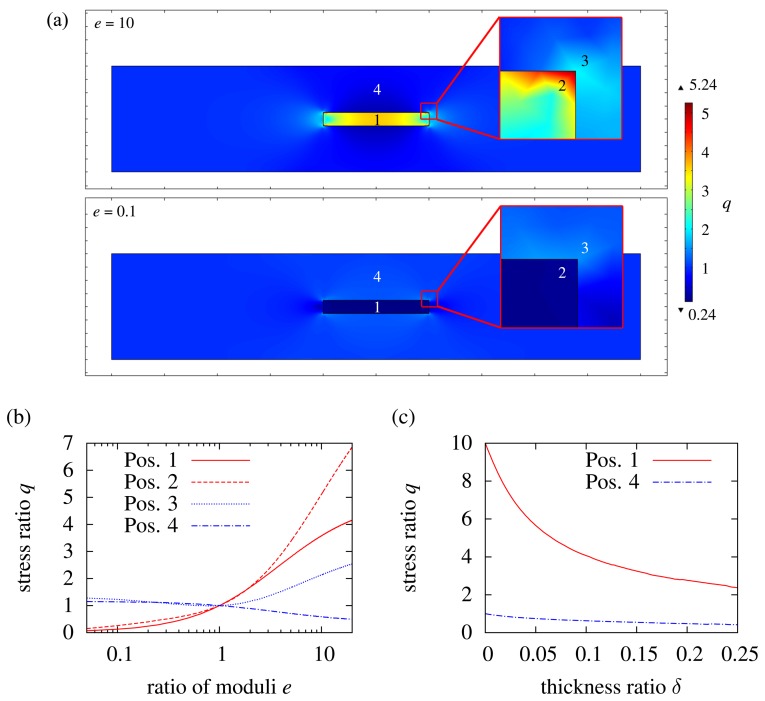
Results of the FEM simulation for tensile load: (**a**) loads in the composite for *e* = 10 and *e* = 0.1; (**b**) load *q* in dependency of **e** at different positions in the composite referring to (a); (**c**) load *q* in dependency of the thickness ratio *δ* for Positions 1 and 4 referring to (a).

The stress ratio *q* at different points in the inlay and in the matrix is dependent on the ratio of the Young's moduli *e*, as shown in Figure 3. In Figure 3b, the inlay is hard with respect to the matrix (*e* = 20). The inlay has to follow the matrix, as considered in Equation (2), and thus, stress is generated in the inlay. The inlay takes a higher load per area than the matrix; this is why stress in the matrix at Position 4 in Figure 3 is reduced. On the edges of the inlay, there is even more stress than in the center of the inlay. This indicates that there is a high risk of delamination at the interface inlay/matrix. An example in this case would be a needle integrated with an elastomer. The needle is not going to follow the movement of the elastomer, but the interface is going to break. In addition to delamination, the high stress at the edges can cause cracks, and due to crack propagation, the matrix fails. If an inlay made of ceramic is integrated with plastic, the edge of the ceramic is pressed into the plastic under external load. The ceramic is more or less like a “dicing-blade”, cutting the plastic from the inside. When the inlay is soft in comparison to the matrix (*e* = 0.05), there is less stress generated in the inlay than in the matrix. The inlay is acting like a hole, reducing the cross-section of the matrix, and thus, the matrix is weakened. Accordingly, the stress in the matrix is increasing when *e* is decreasing, as seen in Figure 3. There is also a load concentration at the edges, because of the sharp rectangular transition. This can cause crack propagation and should be avoided. Compensation techniques therefore will be discussed later in Section 2.3.

To see the influence of the inlay thickness, *d_i_* is varied. To have a more general view of it, the thickness parameter *δ* is used:

(13) $$\delta =\frac{d_{\text{inlay}}}{d_{\text{matrix}}}$$

Figure 3 shows the variation of *δ* between zero and 0.25 for *e* = 10 at the center of the inlay. The thinner the inlay, the higher the stress in it. For *d_i_* → 0, the stress in the inlay is ten-times higher than in the matrix, which is in accordance with the results of Equation (2). If the thickness and, thus, the cross-section of the inlay increases, the inlay takes a higher load. In accordance with this, stress in the matrix is reduced. For a weak inlay, it is the other way round. As we determined in the previous section, a weak inlay in comparison to the matrix is like a hole in the matrix. When the hole gets bigger (*d_i_* increasing), the cross-section of the matrix decreases, while the load remains the same. Thus, higher stress is generated in the matrix.

**Figure 4.**
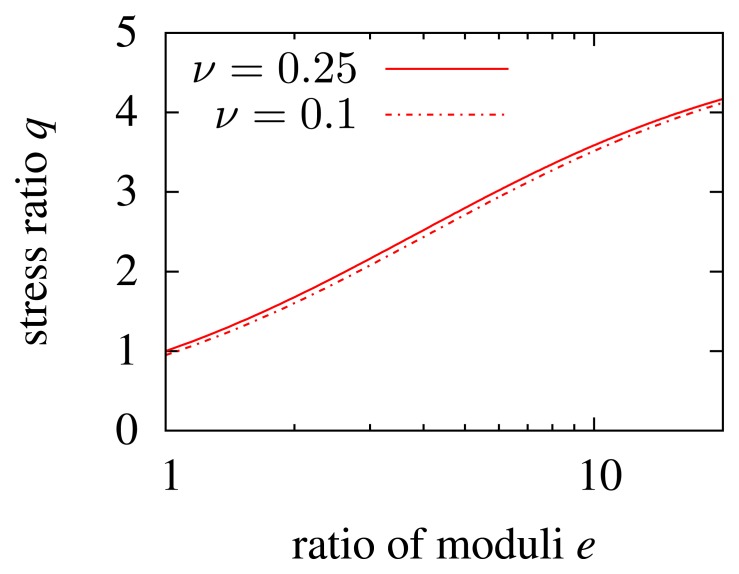
Mechanical load of an inlay at Position 1 referring to Figure 3 in dependency of *e* for two different Poisson ratios: solid line *ν*_i_ = *ν*_m_ = 0.25 (which is the red solid line in Figure 3), dashed line *ν*_i_ = 0.1 and *ν*_m_ = 0.25.

So far, the Poisson ratio of the inlay and matrix is kept constant. To see its influence, the standard geometry from Figure 2 is used, and *ν*_i_ is set to 0.1, while *ν*_m_ = 0.25 is not changed. The result is shown in Figure 4. The mechanical load in the inlay slightly decreases with decreasing Poisson ratio. The lower the Poisson ratio, the higher the rigidity, and thus, the inlay does not contract transversely to a large extent. However, the influence of the Poisson ratio is minimal and can be neglected. The Poisson ratio is directly related to the Young's modulus and, thus, to *e* (the ratio of the moduli). When the Young's modulus of a material increases, the Poisson ratio decreases (rubber ≈ 0.45, plastics ≈ 0.35, metals ≈ 0.25, ceramics ≈ 0.15). The load on a hard inlay would slightly be increased as the Poisson ratio increases. However, as already mentioned, the influence is minimal and does not lead to very low mechanical loads in the inlay for high values of *e*. This is why the Poisson ratio is not taken into consideration any more.

### 2.2. Mechanical Impact of Inlays on the Bending Load

**Figure 5.**
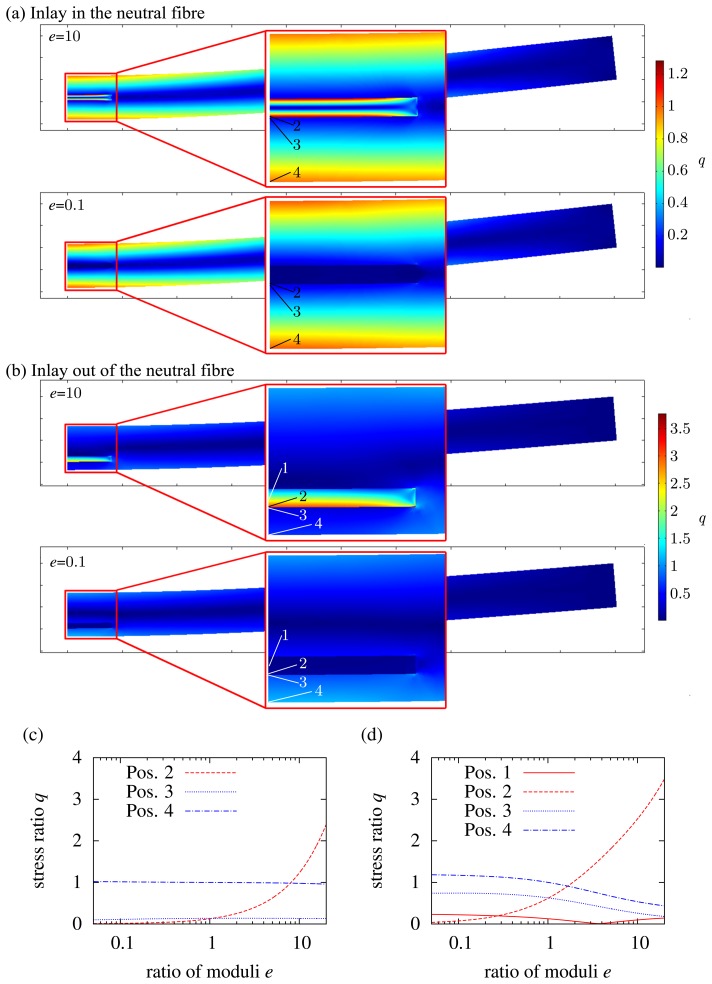
Results of the FEM simulation for the bending load: (**a**,**b**) load in the composite for *e* = 10 and *e* = 0.1; (c) load *q* in dependency of *e* at different positions in the composite referring to (a), inlay in the neutral fiber; (**d**) load *q* in dependency of *e* at different positions in the composite referring to (b), inlay outside the neutral fiber.

The results for an inlay in a neutral fiber are shown in Figure 5a,c. Position 1 is not considered, because it is a neutral fiber, and there is no mechanical load. If we first have a look at a hard inlay, the stress in it is not higher than at the outer edge of the matrix up to *e* = 8. The load at the matrix/inlay interface (Position 4) is low, too. Thus, we can state that an inlay integrated with the neutral fiber can be harder than the matrix up to a certain limit, while it does not downgrade the overall structural behavior. If the inlay is soft, there is nearly no influence. Although the inlay is like a hole in the matrix, it does not downgrade the structure, because it is placed in a neutral fiber, where there is minor mechanical stress. In contrast to this, if an inlay is placed outside the neutral fiber, the influence increases, which is shown in Figure 5b,d. Due to the “hole”, the stress at the outer edges increases by about 20% (*q* = 1.2) with *e* = 0.1. If the inlay is hard with respect to the matrix, the stress at the matrix/inlay interface is much higher than that at the outer edge of the matrix (compare Position 2 and Position 4). The inlay is much more rigid than the inlay, but the elongation of the composite is dominated by the matrix. The hard inlay has the same bending radius the matrix has, while the moment of inertia of the inlay is much higher than that of the matrix. Thus, high stress is generated at the inlay/matrix interface. If the inlay fractures, then there is crack growth, which leads to the failure of the whole composite. One example is a needle in silicone. If the silicone is bent, the needle will not follow the movement of the silicone. The needle will cut through the silicone. Another example is a ceramic in plastics. While the ceramic is much more rigid than the plastic, there is a high risk of cracks in the ceramic. A final conclusion can be made for both types of inlay (hard and soft): sharp edges should be avoided, because there is a high risk of cracks and crack propagation.

**Figure 6.**
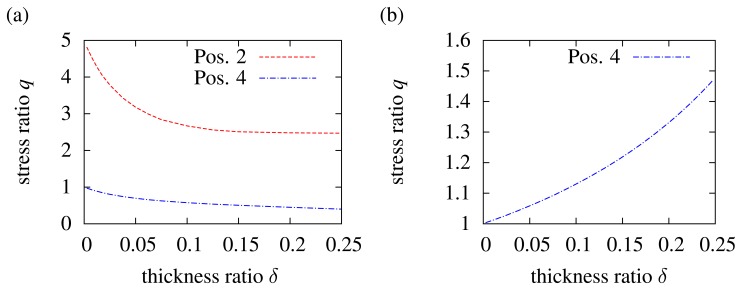
Influence of the inlay thickness for an inlay outside the neutral fiber: (**a**) load factor *q* for a hard inlay (*e* = 10) at Positions 2 (interface inlay/matrix) and 4 (outer edge of the matrix) referring to Figure 5; (**b**) soft inlay (*e* = 0.1) for Position 4 (outer edge of the matrix) referring to Figure 5.

To focus on the influence of the inlay thickness in the case of bending, the thickness parameter *δ* is again used (see Equation (13)). The variation of the inlay thickness in the neutral fiber is not considered. While the neutral fiber has no mechanical load, the load on very thin inlays in the neutral fiber is negligible. This is of course totally different from inlays outside the neutral fiber. Figure 6a shows the variation of *δ* between zero and 0.25 for *e* = 10. If we take a look at the matrix/inlay interface according to Position 2 in Figure 5, the mechanical load increases, while the inlay thickness decreases. A thin and hard inlay does not take a high load in comparison to a thick and hard inlay. Consequently, the mechanical load at the outer edge of the matrix increases, which is seen by the blue dashed line. Of course *q* = 1 is not exceeded, because for *δ* → 0, the inlay thickness becomes zero, and thus, it is equal to a non-existent inlay. Figure 6b shows the variation of *δ* for a soft inlay (*e* = 0.1) at the outer edge of the matrix (in accordance with Position 2 in Figure 5b. Here, it becomes again clear that the inlay is like a hole in the matrix. The thicker the inlay (*δ* increases), the higher is the load at the outer edge of the matrix, because the moment of inertia decreases, while the bending load stays constant.

### 2.3. Structural Design to Reduce Edge Loads

**Figure 7.**
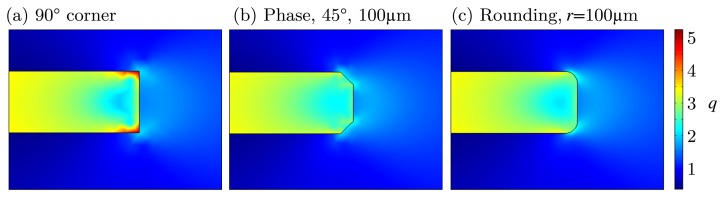
Comparison of edge load for three different types of edges. (**a**) 90° corner (**b**) 45° phase and (**c**) rounding

In some cases, it might not be possible to adapt the inlay to the matrix to reduce mechanical stress caused by a tensile load, for example. The reason for this can be process compatibility, like thermal treatment or less chemical resistance during fabrication. As derived from Figure 3, high stress is generated at the inlay/matrix interface, especially at the edges. While rounding or phasing the edges, the maximum stress is reduced, which can be seen in Figure 7. The basis for this is the standard geometry (see Figure 2). With a 45° phase or a rounding, the maximal load *q*_max_ is reduced to less than 3.5 (in comparison: 90° edge *q* = 5). The results in Section 2.1 have shown that the load in the inlay is *q* = 3.5. Thus, we can state that, due to rounding or phasing of the edges, the highest load is not at the edge anymore, but at the center of the inlay.

## 3. Stress during the Integration Process

In the integration process of the sensor, there will be in most processes thermal treatment of the matrix and, thus, of the sensor. Thermal stress is widely known as:

(14) $$\epsilon_{\text{th}}=\alpha \Delta T$$

with the thermal coefficient of expansion *α* and the temperature difference Δ*T*. Inserting Hooke's law into Equation (3) leads to:

(15) $$\sigma_{\text{th}}=E\alpha \Delta T$$

Due to a mismatch in the thermal coefficients, stress occurs when two materials (which are mechanically connected) are cooled down or heated up. If the inlay is thin in comparison to the matrix and the thickness of the inlay has no influence, then the inlay underlies the elongation during cooling down (or heating up) of the matrix: *∊_i_* = *∊_m_*. If there is a difference in the thermal coefficients of expansion, then *α* = α*_i_ – α_m_*, and thus, Equation (15) is:

(16) $$\sigma_{\text{th}}=E_{i}(\alpha_{i}-\alpha_{m})$$

Thus, the mechanical stress due to integration is in dependencyof the difference of the coefficients of the thermal expansion and the Young's modulus of the inlay. If we think about silicon integrated in aluminum, we would state from the previous section “Mechanical Behavior of an Inlay” that their Young's moduli fit quite well (*E*_Si_ = 130GPa, *E*_al_ = 70GPa), and integration without a high wound effect should be possible. However, there is a large mismatch in the thermal coefficients of elongation: *α*_al_ ≈ 8 × *α*_Si_. Integrating silicon during casting will end in high stress generated in the silicon. In summary, the coefficient of thermal expansion of the inlay has to be adapted to the matrix if there is a significant temperature load during integration. If the stress in the inlay is too high, it might be destroyed or be detached from the matrix, due to tensile or compressive stress.

## 4. Thermal Impact of Inlays

**Figure 8.**
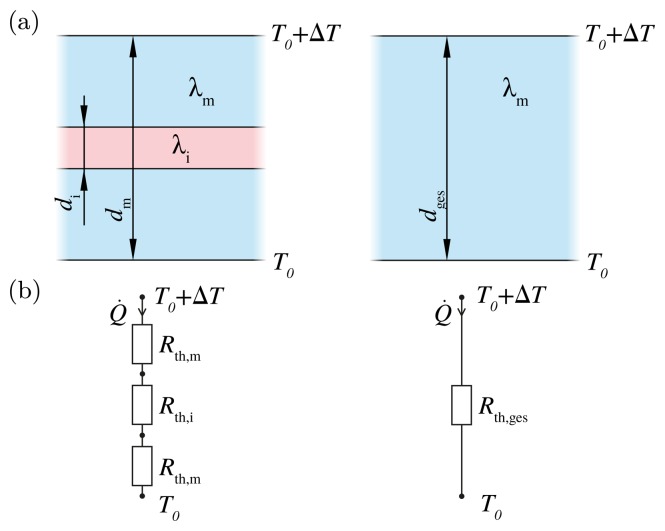
Thermal effect of the wound in a one-dimensional model: (**a**) matrix with and without inlay; (**b**) thermally equivalent circuit.

In parallel to mechanical wound effects, an inlay also has a thermal impact on the matrix when there is a difference between the thermal conductivity of the inlay and matrix. The inlay may act as a thermal barrier or as a thermal bridge. Figure 8 shows again the composite of matrix and inlay with the thickness *d_m_* and *d_i_* and thermal conductivity λ*_m_* and λ*_i_*. On one side, the temperature is higher by Δ*T*. Thus, there is a heat flow *Q*, which can be calculated by the thermal resistance *R*_th_ and the temperature difference Δ*T*:

(17) $$\dot{Q}=\frac{\Delta T}{R_{\text{th}}}$$

Let us assume that the area of the inlay and matrix are equal and that we have a one-dimensional problem. Then, the structure can be replaced by an equivalent circuit. The thermal resistance *R*_th_ is the thickness *d* divided by the product of the cross-section *A* and the material-dependent thermal conductivity λ:

(18) $$R_{\text{th}}=\frac{d}{\lambda \times A}$$

With an inlay, the thermal resistivity is calculated as follows:

(19) $$\begin{array}{c} R_{\text{th},\text{ges}}=\frac{d_{m}-d_{i}}{2\times \lambda_{m}\times A}+\frac{d_{i}}{\lambda_{i}\times A}+\frac{d_{m}-d_{i}}{2\times \lambda_{m}\times A}\\ R_{\text{th},\text{ges}}=\frac{d_{m}-d_{i}}{\lambda_{m}\times A}+\frac{d_{i}}{\lambda_{i}\times A} \end{array}$$

The cross-section of the inlay and matrix are equal. Thus, we can regard the thermal resistance of the area *R*_th_ = *R*_th_ × *A*. Then, Equation (19) becomes:

(20) $$R_{\text{th}}=\frac{d_{m}-d_{i}}{\lambda_{m}}+\frac{d_{i}}{\lambda_{i}}$$

If we think about the inlay thickness, which is 0.1-times the matrix thickness, but the thermal conductivity of the inlay is ten-times higher in comparison to the matrix, then the thermal resistance of the area is nearly doubled, according to Equation (20) (exact value: 1.9). If the inlay thickness decreases and runs against zero, the influence of the inlay is negligible, which can be seen by Equation (20).

The one-dimensional approach can give a simple approximation of how the thermal wound effect dominates the overall behavior. To give an illustration, the standard geometry used for tensile load is investigated in the thermal behavior. The lower side of the matrix is set to *T*_0_, while the upper side is set to *T*_0_ + Δ*T*. The solution was derived with Comsol Multiphysics using the heat transfer in solids module and a stationary study. The average mesh quality was kept higher than 0.8.

To have a more suitable view of things, the dimensionless temperature factor *μ* with local temperature *T* is used:

(21) $$\mu =\frac{T-T_{0}}{\Delta T}$$

**Figure 9.**
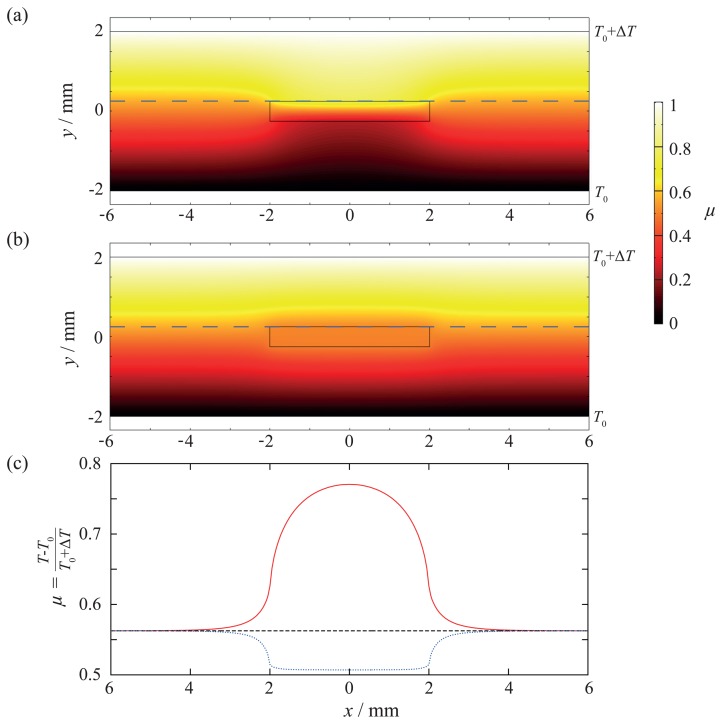
FEM simulation to show the thermal impact of the inlay: (**a**) λ*_i_* = 0.1 λ*_m_ →* heat bridge; (**b**) λ*_i_* = 10λ*_m_ →* heat barrier; (**c**) thermal load factor *μ* along the blue dashed line in (**a**) and (**b**): red solid, λ*_i_* = 0.1λ*_m_*; blue dotted, λ*_i_* = 10λ*_m_*; black dashed, no inlay.

Figure 9a shows the result for a ten-times lower (λ*_i_* = 0.1 λ*_m_*) thermal conductivity of the inlay. The temperature factor *μ* along the x-axis above the inlay is shown in Figure 9c. A thermal impact due to the inlay can be clearly seen. Due to the lower thermal conductivity, a hot spot is created. Without the inlay, the temperature coefficient above the inlay would be *μ* = 0.56, but with inlay, it increases to *μ* = 0.77. This has three consequences: First, overheating can lead to failure of the matrix, because the maximal working temperature is exceeded. Second, a sensor that is sensitive to temperature is measuring the hot spot temperature, but not the real temperature. Third, this can lead to thermally-induced mechanical stress if there is a mismatch in the thermal coefficients of expansion of the inlay and matrix, which might be the reason for mechanical failure (see also Section 3). The results for an inlay with a ten-times higher thermal conductivity (λ*_i_* = 10λ*_m_*) are shown in Figure 9b,c. In this case, instead of a hot spot, a heat bridge is created. Overheating cannot occur, but a sensor that is sensitive to temperature will not measure the right temperature. Additionally, in case there is a mismatch in the thermal coefficients of expansion, thermally-induced mechanical stress will occur, as well. Therefore, it can be determined that a lower or higher thermal conductivity of the inlay can lead to different failures.

The thermal wound effect can be reduced in two ways: on the one hand, the inlay can be made as thin as possible; on the other hand, the thermal conductivity of the inlay has to be adapted to the matrix. However, in this case, the thermal coefficients have to be adapted, too, to reduce or avoid thermally-induced mechanical stress.

## 5. Further Foreign Body Effects

The wound effects presented in the last section are the most common ones, because construction parts are always treated mechanically and/or exposed to temperature changes. Besides, there are, of course, more wound effects than these mentioned above. If we think about moisture, an inlay can be an obstacle by behaving like a moisture barrier in parallel to overheating in thermal aspects (see Section 4). In addition, the inlay and matrix show different behaviors when exposed to radiation. If we think about UV rays, there might be the risk of cracks if the inlay is not resistant to UV rays. The electrical connection of the sensor has not been focused on yet. Wires are also a wound effect. Integrating wireless systems in plastics is not a problem, but this will not work for metals.

The aging behaviors of the inlay and matrix might be different, as well. This is one of the most critical aspects when we talk about long-term material integrated sensors. If the sensor fails, but not the matrix, this has to be detected. Due to friction or diffusion at the inlay/matrix interface, the inlay can be detached from the matrix. Thus, the sensor changes its characteristic signal, which can be misinterpreted as a change of the matrix.

The material strength under various aspects has to be taken into account, too. In case of mechanical load, the inlay needs the same as or a higher material strength than the matrix. The same applies to temperature treatment: the inlay needs the same as or a higher temperature resistance than the matrix. On the one hand, the composite of the matrix and inlay is not that resilient, but a matrix would be without an inlay if these aspects were not taken into account. On the other hand, an inlay can be placed at a position where the maximal resilience is absent, but sufficient sensitivity for generating the desired measurement quantities in the material is still present.

## 6. Fabrication of Material-Integrated Sensors

**Figure 10.**
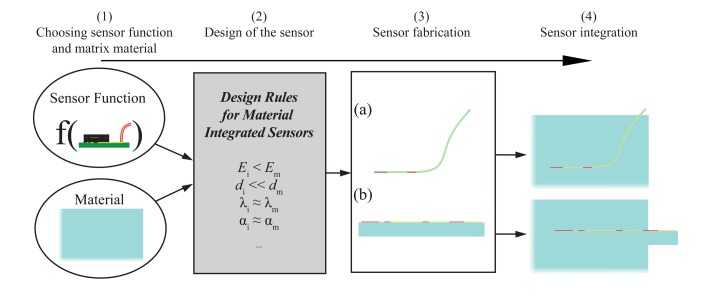
Process flow for material-integrated sensors: (1) The sensor function and the matrix material, in which the sensor will be integrated, are chosen. (2) Designing the inlay based on the rules, which are derived from the results of the FEM study. (3) Fabrication of the sensor in two different ways: (**a**) thin and flexible foil with *d* < 10 μm or (**b**) using the matrix material as the substrate. (4) Integration of the inlay in the matrix; the substrate is merged together with the matrix and, thus, not recognizable.

While in the last sections, we focused on the impact of the inlay on the matrix, we want to present now our idea for the fabrication of material-integrated sensors. Two approaches for how to integrate sensors in a material with established technologies have been presented at the beginning (see Figure 1). From the results, we can see that there is a “wound effect” for these approaches, which changes the macroscopic behavior. To bring this wound effect to a minimum, material-integrated sensors have to be built up in a new way, which is called function scale integration [34]. Figure 10 shows a general workflow for material-integrated sensors. Based on the knowledge derived from modeling, specific design properties can be given for different material systems. We want to explain this for two examples. The goal is to integrate a strain gauge in CFRP and aluminum:

- Carbon fiber-reinforced polymer: Silicon integrated in CFRP will lead to high stress at the edges, because it is harder than CFRP. Furthermore, it is brittle, and a single crystal can quickly lead to cracks in the inlay and delamination during mechanical load. Thus, a strain gauge integrated with CFRP has to be build up as a thin and flexible foil, as shown in Figure 10a. The thickness of a single carbon fiber is around 7 μm. Thus, the sensor thickness should be less than 10 μm. For handling, a standard silicon wafer can be used to fabricate the foil, which is then peeled from the silicon. The foil can be made out of high temperature, stable plastic, like polyimide, which can withstand 180 degree during the fabrication of the CFRP. There is still the remaining danger of delamination, because the sensor is an “area of discontinuity”. Thus, good adhesion at the interface matrix/inlay is necessary. This can be improved by holes in the foil to let the resin flow through [34]. For the conductor path, soft metals, like gold or aluminum, can be used, which have a similar Young's modulus as the CFRP.
- Aluminum: High stress is generated when silicon is integrated with aluminum during casting, because of the large difference in the coefficients of thermal expansion. Furthermore, silicon is brittle, and high compressive strain will crush it. A foil will burst in the flow of liquid aluminum at a pressure up to 300 bar. The best solution is to build up the sensor on an aluminum substrate (e.g., *d* = 0.8 mm), as shown in Figure 10b. After integration, the substrate cannot be recognized, because it is merged with the matrix. The most challenging part will be the isolation layer. Standard ceramics used in MEMS technology, like silicon dioxide or nitride, have a ten-times lower coefficient of thermal expansion. This will lead to high compressive stress, and there is the danger that it will dissolve away from the substrate. Zirconium oxide, for example, has a two-times lower coefficient of thermal expansion. This will lead to less stress than silicon oxide or nitride, but compressive stress during integration cannot be avoided. The conductor path can be made of aluminum, which will, of course, not be a wound effect, because aluminum is also the matrix material.

Thus, we see that integrating sensors in a material will only be possible when the design of the sensor is adapted to the integration process with the matrix. Consequently the sensors and the integration process have to be as minimally invasive as possible, to reduce the wound effect to dimensions of other material defects, like entrapped air or impurities.

## 7. Conclusions

We investigate the question “What is the impact of the inlay on the matrix?” for material-integrated sensors by using the finite element method. This was done by looking at the behavior of an inlay in the matrix under mechanical, thermal, thermo-mechanical and other loads with alternating geometries. For inlays treated under mechanical load, we can state that the inlay has to be at least as stretchable and bendable as the matrix, because comparatively hard inlays lead to a high wound effect. However, these inlays should not be too thick with respect to the matrix, because a relatively thick and soft inlay can weaken the inlay. During integration, inlays can be set under high mechanical load if the coefficients of thermal expansion differ strongly. Consequently, the thermal expansion of the inlay has to be adapted to the matrix, if there is a high thermal load during integration. In the case of a high thermal load, the inlay has to be as thin as possible or its thermal conductivity has to be adapted to the thermal conductivity of the matrix. On the derived results, we presented our idea for the fabrication of sensors adapted to the matrix material and the integration process.

In future work, we will manufacture material-integrated sensors on the basis of the theoretical investigations made in this article. Taking mechanical, thermal and other measurements, investigations on the effect of the wound have to be made. Furthermore, the long-term stability of material-integrated sensors has to be analyzed in theoretical aspects and, of course, in experiments by doing cycling loading tests, for example.
